# The ATP/Mg^2+^ Balance Affects the Degradation of Short Fluorogenic Substrates by the 20S Proteasome

**DOI:** 10.3390/mps5010015

**Published:** 2022-02-05

**Authors:** Alexey Morozov, Tatyana Astakhova, Pavel Erokhov, Vadim Karpov

**Affiliations:** 1 Laboratory of Regulation of Intracellular Proteolysis, Engelhardt Institute of Molecular Biology, Russian Academy of Sciences, 119991 Moscow, Russia; karpov@eimb.ru; 2 Laboratory of Biochemistry, Koltzov Institute of Developmental Biology, Russian Academy of Sciences, 119334 Moscow, Russia; tastakhova@bk.ru (T.A.); paer@gmx.com (P.E.)

**Keywords:** proteasome, immunoproteasome, proteasome activity, fluorogenic substrates

## Abstract

Proteasomes hydrolyze most cellular proteins. The standard reaction to determine proteasome activity in cellular lysate or elsewhere contains AMC-conjugated peptide substrate, ATP, Mg^2+^, and DTT. ATP and Mg^2+^ are included to maintain 26S proteasome functionality. However, most cellular proteasomes are 20S proteasomes, and the effects of ATP on the turnover of fluorogenic substrates by 20S complexes are largely unknown. Here, we evaluated the effect of ATP alone or in combination with Mg^2+^ on the degradation of AMC-conjugated fluorogenic substrates by purified 20S proteasomes. Degradation of substrates used to determine chymotrypsin-, caspase- and trypsin-like proteasome activities was gradually decreased with the rise of ATP concentration from 0.25 to 10 mM. These effects were not associated with the blockage of the proteasome catalytic subunit active sites or unspecific alterations of AMC fluorescence by the ATP. However, ATP-induced peptide degradation slowdown was rescued by the addition of Mg^2+^. Moreover, the substrate degradation efficacy was proportional to the Mg^2+^/ATP ratio, being equal to control values when equimolar concentrations of the molecules were used. The obtained results indicate that when proteasome activity is assessed, the reciprocal effects of ATP and Mg^2+^ on the hydrolysis of AMC-conjugated fluorogenic substrates by the 20S proteasomes should be considered.

## 1. Introduction

Cells have two systems responsible for the destruction of organelles and proteins. Entire organelles and long-lived proteins are degraded by autophagy, while most intracellular short-lived proteins are hydrolyzed by the ubiquitin–proteasome system (UPS) [1]. Within the UPS, the proteasomes perform degradation of protein substrates. The 26S proteasome is capable of selective recognition and degradation of ubiquitinated proteins. It is composed of a 19S regulator and a 20S core particle. The 19S complex allows specific recognition and ATP-dependent unfolding and translocation of the substrates into the 20S proteasome, where the protein hydrolysis takes place [2]. The 20S proteasome is a multisubunit protein complex capable of hydrolyzing peptide bonds after acidic, basic, and hydrophobic amino acids. Thus, the proteasome has caspase-, trypsin- and chymotrypsin-like proteolytic activities. Due to their function, proteasomes are involved in almost all cellular metabolic processes and maintain proteostasis in normal and stress conditions. Therefore, proteasome activity is tightly regulated and represents an important physiological parameter [3]. The proteasome activity is frequently assayed in the cellular homogenate which contains both 26S and 20S proteasomes. The classical assay mixture is a buffered solution containing short fluorogenic peptide substrates, DTT, ATP, and Mg^2+^, which are necessary to maintain 26S proteasome function and integrity [4]. The 26S proteasome is an ATP-dependent protease, and consequently, the effects of ATP on its activity have been thoroughly investigated [5,6,7,8]. However, the predominant form of intracellular proteasomes is the 20S proteasome [9], and the effects of ATP on the degradation of peptide substrates by these proteasomes are poorly known. Here, we investigated the effects of ATP on the degradation of short fluorogenic substrates by purified 20S proteasomes.

## 2. Materials and Methods

### 2.1. Proteasome Activity Measurements

Proteasome activities were measured as described in [10]. In brief, aliquots containing 100 ng of purified human constitutive and immune 20S proteasomes (both from Enzo, Farmingdale, NY, USA) were mixed with 90 µL of the reaction buffer (20 mM Tris-HCl (pH 7.5), 1 mM DTT, and 30 µM of substrate), supplemented with different concentrations of ATP (either Sigma Aldrich, Saint Louis, MO, USA or Thermo Scientific, Waltham, MA, USA) and/or Mg^2+^ (Merck, Kenilworth, NJ, USA) and incubated for 20 min at 37 °C. In addition, constitutive 20S proteasomes were incubated with several concentrations of reduced (GSH) or oxidized glutathione (GSSG), or their mixture (both from AppliChem, Darmstadt, Germany). For the determination of chymotrypsin-, caspase- and trypsin-like proteasome activities: Suc-LLVY-AMC (Sigma Aldrich, Saint Louis, MO, USA), Z-LLE-AMC (Sigma Aldrich, Saint Louis, MO, USA), and Ac-RLR-AMC (Enzo, Farmingdale, NY, USA) were used, respectively. Reactions were stopped with 2% SDS solution and fluorescence (excitation at 380 nm) was measured at 440 nm using Versa Fluor Fluorometer (Bio-Rad, Hercules, CA, USA).

### 2.2. Determination of Proteasome Activities in Native Gel

Constitutive 20S proteasome preparations (0.5 µg/track) (Enzo, Farmingdale, NY, USA) were loaded onto the 4-20% gradient polyacrylamide gel. The electrophoresis was performed for 36 h at +4 °C (at 80 V 12 h, 140 V 12 h, and 240 V 12 h). Following the electrophoresis, the gel was cut into slices. Then gel slices containing proteasome preparations were soaked with 6 mM of ATP or 3, 6 or 20 mM of Mg^2+^, or their combinations, together with 100 µM of Suc-LLVY-AMC substrate. After that, gel slices were incubated for 30 min and analyzed under UV. ImageJ software (https://imagej.net/software/fiji/ accessed on 12 June 2020) was used to analyze the acquired data. Gels were stained with ROTI^®^Blue quick protein stain (Carl Roth, Karlsruhe, Germany) to confirm an equal amount of proteasomes.

### 2.3. Determination of Proteasome Activity Using Proteasome Activity-Based Probe

The Me4BodipyFL-Ahx3Leu3VS— cell-permeable proteasome activity-based probe (UbiQbio, Amsterdam, The Netherlands) was used to estimate the activity of proteasomes after the incubation with different concentrations of ATP and Mg^2+^. Preparations of purified constitutive 20S proteasomes (0.25 µg) (Enzo, Farmingdale, NY, USA) were incubated with 1 µM of the probe and either PBS, or 1, 6 and 10 mM of ATP, or 1, 6, and 20 mM of Mg^2+^, or with 6 mM of ATP and 6 mM of Mg^2+^ during 1.5 h at 37 °C. After that, samples were mixed with the 2x SDS PAGE Sample buffer (Invitrogen, Carlsbad, CA, USA), incubated for 10 min at 95 °C, and loaded onto the 13% PAG. Following the electrophoresis, the gel was analyzed using the ChemiDoc XRS+ imaging system (Bio-Rad, Hercules, CA, USA) with filters allowing excitation at 480 nm and emission at 530 nm in accordance with [11].

### 2.4. Evaluation of AMC Fluorescence in the Presence of Different ATP Concentrations

Here, 5 µM 7-amido-4-methylcoumarin AMC (Acros Organics, Geel, Belgium) was incubated in the reaction buffer containing 50 mM Tris-HCl (pH 7.5), 100 mM NaCl supplemented with 0, 1, 5, and 10 mM of ATP, with or without 2 mM of Mg^2+^ for 20 min at 37 °C. The AMC fluorescence was estimated as the endpoint measurements at the excitation wavelength 380 nm and emission wavelength 440 nm using Versa Fluor Fluorometer (Bio-Rad, Hercules, CA, USA).

### 2.5. Statistics

The experiments with statistical analysis were performed at least in triplicate. Bar charts depict mean values ± standard deviation. An unpaired two-tailed *t*-test was used to evaluate the statistical significance. *p*-values less than 0.05 were regarded as statistically significant. Asterisks indicate: * *p* < 0.05; ** *p* < 0.01; *** *p* < 0.001; **** *p* < 0.0001. *p*-value calculations were performed using GraphPad Prism version 8.4.3. (GraphPad Software, San Diego, CA, USA; RRID:SCR_002798) software. Trend lines were built using the Microsoft Office application (Microsoft corp., Redmond, WA, USA).

## 3. Results and Discussion

**Figure 1:**
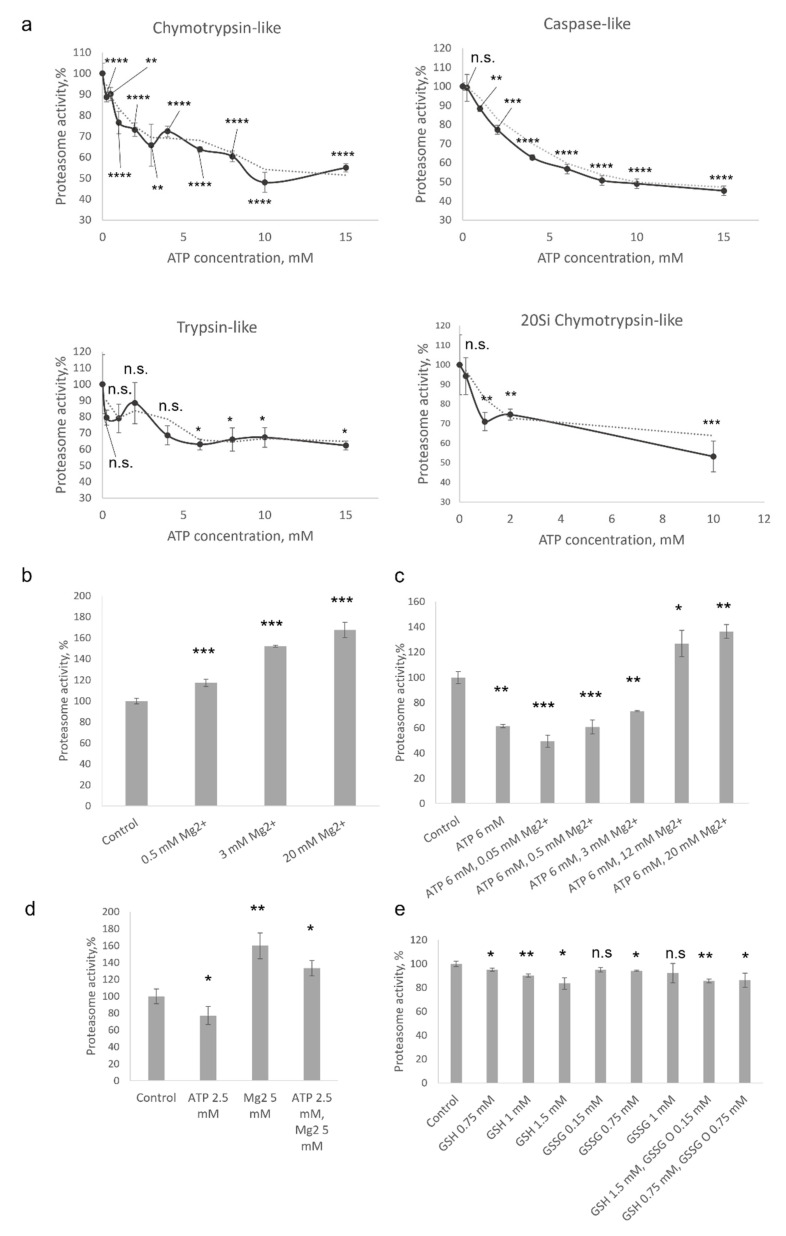
Reciprocal effects of ATP and Mg^2+^ on the degradation of fluorogenic peptides by 20S proteasomes. (**a**) The chymotrypsin-, caspase- and trypsin-like activities were determined using Suc-LLVY-AMC, Z-LLE-AMC, and Ac-RLR-AMC fluorogenic peptides, respectively, as substrates. First, 100 ng of constitutive or immune (indicated as 20Si) 20S proteasomes was used in the reactions, mixed with 90 µL of the reaction buffer (20 mM Tris-HCl (pH 7.5), 1 mM DTT, and 30 µM of substrate), supplemented with indicated concentrations of ATP. (**b**) The degradation of Suc-LLVY-AMC (chymotrypsin-like activity) by constitutive 20S proteasomes in the presence of different concentrations of Mg^2+^. (**c**) The degradation of Suc-LLVY-AMC (chymotrypsin-like activity) by constitutive 20S proteasomes in the presence of 6 mM of ATP and different concentrations of Mg^2+^. (**d**) The degradation of Suc-LLVY-AMC (chymotrypsin-like activity) by constitutive 20S proteasomes in the presence of 2.5 mM of ATP and 5 mM of Mg^2+^ (**e**) The chymotrypsin-like activity of 20S proteasomes in the presence of different concentrations of reduced (GSH) or oxidized (GSSG) glutathione. The proteasome activity in the control samples is indicated as 100%. The experiments with statistical analysis were performed at least in triplicate. Bar charts depict mean values ± standard deviation. An unpaired two-tailed *t*-test was used to evaluate the statistical significance. *p*-values less than 0.05 were regarded as statistically significant. Asterisks indicate * *p* < 0.05; ** *p* < 0.01; *** *p* < 0.001; **** *p* < 0.0001. *p*-value calculations were performed using GraphPad Prism version 8.4.3. (GraphPad Software, San Diego, CA, USA; RRID:SCR_002798) software. Dotted curves represent trend lines. Trend lines were built using the Microsoft Office application (Microsoft corp., Redmond, WA, USA).

The assay based on the degradation of short peptides conjugated to 7-amido-4-methylcoumarin (AMC) has several limitations [12]; nevertheless, such peptides are the most widely used substrates for proteasome activity measurements [13]. Here, we studied the effect of ATP on the 20S proteasome-mediated degradation of Suc-LLVY-AMC, Z-LLE-AMC, and Ac-RLR-AMC peptide substrates used to evaluate chymotrypsin-, caspase- and trypsin-like proteasome activities, respectively. It has been shown that the degradation of all three substrates by the 20S proteasome was dose-dependently attenuated in the presence of ATP (Figure 1a). The degradation of Suc-LLVY-AMC was reduced by 10% when 0.25 mM of ATP (*p* < 0.0001, *t*-test) was present in the reaction mixture, but reached 50% of the control values when ATP concentration was 10 or 15 mM (*p* < 0.0001, *t*-test). Common kinetics was revealed with the caspase-like-activity substrate Z-LLE-AMC. The degradation of Ac-RLR-AMC was slightly less affected, and the values revealed in samples incubated with 10–15 mM of ATP were 35–40% (*p* < 0.05, *t*-test) lower than in controls (Figure 1a). In addition, ATP significantly and dose-dependently decreased the efficacy of chymotrypsin-like-activity substrate degradation by the 20S immunoproteasomes (*p* < 0.001, *t*-test at ATP concentration 10 mM) (Figure 1a). Immunoproteasome is a specific form of proteasome found mostly in the cells of the immune system or upregulated in somatic cells because of stimulation by inflammatory cytokines or during stresses [3]. Finally, the effect of 1 mM of DTT was tested; no influence on the activity of proteasomes was revealed (data not shown). Thus, our results indicated that the degradation of short substrates by the 20S proteasome might be modulated by the ATP molecules. Here, it should be mentioned that no effect of 10 µM to 5 mM of ATP on the degradation of Suc-LLVY-AMC by 20S proteasomes has been reported by Geng et al. [5]. The discrepancy between these data and our results might be associated with different concentrations of proteasomes in the reaction: 4 μg/mL in [5] and 1 µg/mL in our experiments, as well as the constant presence of 5 mM of magnesium in the reaction mixtures described by Geng et al. [5].

The intracellular concentration of ATP is in the low millimolar range mostly between 0.5 and 5 mM, depending on a cell type in physiological conditions [14]. However, most of the cellular ATP is associated with Mg^2+^ ions. Interestingly, several divalent ions including Ca^2+^ and Mg^2+^ have been shown to modulate proteasome activity [15,16]. Magnesium ions facilitate the degradation of β-casein [17]. By using fluorogenic peptides, it has been shown that the caspase-like activity of bovine proteasome increased by Mg^2+^, while trypsin-like activity and chymotrypsin-like activity decreased [17]. Moreover, Mg^2+^ ions have been reported to enhance the degradation of casein by archaeal 20S proteasome [18]. The authors demonstrated increased turnover of a fluorogenic peptide by the 20S proteasome in the presence of magnesium and suggested that it is associated with improved binding and catalysis [18]. At the same time, concentrations of magnesium (25–500 mM) much higher than physiological concentration were used. Thus, in a more recent paper, a 2–3-fold increase in fluorogenic peptide degradation by the archaeal proteasomes in the presence of physiological concentrations (1–5 mM) of Mg^2+^ was demonstrated [16]. At the same time, no effect of Mg^2+^ ions on the proteasome activity was shown by another group [5]. Taken together a certain contradiction in the reported data can be observed. Therefore, and since ATP is mainly complexed with magnesium ions, which can influence the proteasome activity, we evaluated the effect of Mg^2+^ on the degradation of fluorogenic peptides by the constitutive 20S proteasomes.

Magnesium ions dose-dependently stimulated the degradation of Suc-LLVY-AMC by constitutive 20S proteasomes (Figure 1b). Proteasome activation from 17% to 67% was observed when Mg^2+^ concentrations from 0.5 mM to 20 mM were used, correspondingly (*p* < 0.001, *t*-test). In the next experiment, the degradation of Suc-LLVY-AMC by the 20S proteasomes in the presence of both ATP and Mg^2+^ ions was evaluated. A fixed concentration of ATP − 6 mM and varying concentrations of Mg^2+^ from 0.05 mM to 20 mM were used. As expected, when 20S proteasomes were incubated with ATP only, a significant decrease (60% from control values) in substrate turnover was observed (*p* < 0.001, *t*-test); however, the degradation rate began to rise when Mg^2+^ concentrations were more than 0.5 mM (Figure 1c). The activity reached control values when equimolar concentrations of ATP and Mg^2+^ were used and continued to rise with the concentration of magnesium in the reaction (Figure 1c). The compensatory effect of Mg^2+^ on the ATP-stimulated decrease of peptide degradation is evident when samples incubated with 20 mM of Mg^2+^, and with and without 6 mM ATP, are compared (Figure 1b,c). Additionally, we studied the combined and separate effects of commonly used concentrations of ATP and of Mg^2+^. A 33% higher chymotrypsin-like activity was observed when 2.5 mM of ATP and 5 mM of Mg^2+^ were present in the reaction (Figure 1d).

Thus, we assume that a larger amount of proteasomes in the experiments [5] and the presence of relatively high concentration (compared to ATP) of magnesium might buffer the effect of ATP on the degradation of the substrate by the 20S proteasomes, explaining the difference between our results and data reported by Geng and coauthors [5].

To estimate how other small molecules affect 20S proteasome activity, we used glutathione—the most abundant low-molecular-weight thiol compound synthesized in cells [19]. Glutathione maintains redox homeostasis and protects cells from oxidative damage. A reaction mixture containing 100 ng of constitutive 20S proteasomes and Suc-LLVY-AMC substrate was supplemented with different concentrations of reduced (GSH) or oxidized glutathione (GSSG), or their mixtures. Physiological concentrations of reduced (GSH) and oxidized glutathione (GSSG), which is increased in stress conditions, were used. It has been shown that with the increase of GSH concentration, the efficacy of substrate degradation by 20S proteasomes decreases, although to a lesser extent than in the case of equimolar concentrations of ATP (Figure 1a,e).

**Figure 2:**
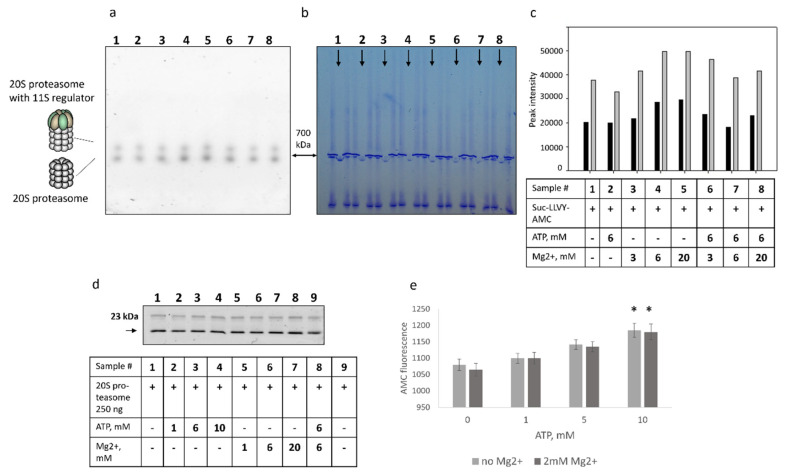
The effect of free ATP on the degradation of the Suc-LLVY-AMC fluorogenic peptide by the proteasomes in the Native gel. Decreased fluorogenic peptide degradation in the presence of ATP is not associated with the blockage of the active sites of catalytic subunits and is independent of the association of the ATP with the AMC. (**a**) The 500 ng of constitutive 20S proteasome were loaded onto the Native 4–20% gradient gel. Tracks containing proteasomes (indicated by arrowheads) were separated by two tracks with the reference proteins thyroglobulin ~670 kDa and ferritin ~440 kDa. Gel areas with proteasomes were soaked in solutions containing: (1) a 100 µM of Suc-LLVY-AMC substrate; (2) a 100 µM of Suc-LLVY-AMC substrate and 6 mM of ATP; (3) a 100 µM of Suc-LLVY-AMC substrate and 3 mM of Mg^2+^; (4) a 100 µM of Suc-LLVY-AMC substrate and 6 mM of Mg^2+^; (5) a 100 µM of Suc-LLVY-AMC substrate and 20 mM of Mg^2+^; (6) a 100 µM of Suc-LLVY-AMC substrate, 6 mM of ATP and 3 mM of Mg^2+^; (7) a 100 µM of Suc-LLVY-AMC substrate, 6mM of ATP and 6 mM of Mg^2+^; (8) a 100 µM of Suc-LLVY-AMC substrate, 6 mM of ATP and 20 mM of Mg^2+^ and analyzed under UV. (**b**) The same gel was stained with ROTI^®^Blue quick protein stain. (**c**) Analysis of image A using ImageJ software. The intensities of peaks with larger molecular weight are shown in dark grey, the intensities of bands with 700 kDa molecular weight are shown in light grey. A data table is given below the graph. (**d**) Preparations of purified constitutive 20S proteasomes (0.25 µg) were incubated with 1 µM of the Me4BodipyFL-Ahx3Leu3VS proteasome activity probe in the presence or absence of the ATP and Mg^2+^ (indicated in the Table) for 1.5 h at 37 °C. After that, samples were mixed with the 2 × SDS PAGE Sample buffer incubated for 10 min at 95 °C and loaded onto the 13% PAG. Following the electrophoresis, the gel was analyzed in the fluorescent imager. (**e**) Dependence of the AMC fluorescence on the concentration of ATP. Here, 5 µM of AMC were incubated with different concentrations of ATP, and the AMC fluorescence was measured at the excitation wavelength 380 nm and emission wavelength 440 nm. Bars represent standard deviation (*n* = 3). An unpaired two-tailed *t*-test was used to evaluate the statistical significance. Asterisks indicate * *p* < 0.05. *p*-value calculations were performed using GraphPad Prism version 8.4.3. (GraphPad Software, San Diego, CA, USA; RRID:SCR_002798) software.

Next, we investigated the effect of both ATP and Mg^2+^ ions on the 20S proteasome preparations in the Native gel. Commercial 20S proteasome preparations revealed two forms of proteasomes with distinct molecular weights—700 kDa and 800–900 kDa, respectively. Based on previous findings by us and others and Western blot analysis (not shown), the heavier form represented 20S proteasome with 11S activator. Hence, we sought to evaluate if the effect of ATP and Mg^2+^ on these two proteasome forms is different. The obtained results (Figure 2a–c) revealed separate and mutual effects of ATP and Mg^2+^ on the degradation of fluorogenic substrates by the 20S proteasomes (Figure 1b–d). The activity of the 20S proteasome with the 11S regulator followed the same dynamics as the activity of the 20S proteasome, though with the lesser amplitude, indicating that ATP/Mg^2+^ influences the turnover of fluorogenic peptides by both proteasome forms.

To test if ATP or Mg^2+^ affect the accessibility of the catalytic subunit active sites, we used the proteasome activity-based probe Me4BodipyFL-Ahx3Leu3VS. No significant differences in proteasome subunit fluorescence were revealed (Figure 2d). Although one cannot exclude that binding of the probe to the catalytic proteasome subunits might be attenuated in the presence of ATP, the obtained results indicate that ATP and Mg^2+^ likely have minimal effect on the interaction of the probe with the active sites of the catalytic subunits. These results raised a possibility that decreased AMC fluorescence after the incubation of 20S proteasomes with the ATP (Figure 1) might be associated with the interaction of ATP with the AMC, leading to a decreased AMC fluorescence. To address this issue, we incubated the free AMC with different concentrations of ATP. Interestingly, a modest ATP concentration-dependent increase of AMC fluorescence was observed (*p* < 0.05, *t*-test at the ATP concentration of 10 mM) (Figure 2e). Thus, the obtained results indicated that decreased proteasome activity in the presence of ATP is likely not associated with the blockage of the active sites of catalytic subunits and is independent of quenching of the AMC fluorescence by the ATP.

In cells, the amount of free ATP can rise in certain conditions [20], although generally the ATP is complexed with magnesium ions. Our results and data reported previously [14,15,16] indicated that Mg^2+^ increases the efficacy of proteasome-dependent substrate turnover. We showed that when 20S proteasomes were incubated with free ATP, the substrate degradation efficacy decreased, but were restored following the addition of an equimolar concentration of Mg^2+^ (Figure 1c). Moreover, our data indicated that the degradation of substrates by 20S proteasomes with 11S regulators is likewise influenced by ATP and Mg^2+^. These results are essential for the selection of ATP and Mg^2+^ concentrations in the AMC-conjugated peptide substrate-based reactions used to evaluate proteasome activity in cellular lysates. The significance of the obtained data beyond these experimental settings should be addressed further. If the balance in the compound concentration affects larger proteasome substrate turnover by the 20S proteasomes, it might be relevant, for example, in stress conditions. During stress, Mg^2+^ concentrations are decreased, while the amount of free ATP may increase [20]. In stress conditions, 20S proteasomes (constitutive and immune) and 20S proteasomes with 11S complexes are indispensable for the degradation of oxidized and damaged proteins [21]. Moreover, modulation of proteasome substrate turnover by ATP/Mg^2+^ balance may influence basic cellular metabolic processes like respiration [20] and mitosis [22], affect the production of antigenic peptides used for presentation in complexes with MHC-I leading to the altered immunological responses. Furthermore, the blood ATP/Mg^2+^ ratio can modulate the activity of 20S proteasomes circulating in the blood. Since the 20S proteasome is the only proteasome form found in blood [23], a small molecule ratio-based regulatory mechanism may be essential for the proteostasis of the entire organism. Indeed, changes in extracellular proteasome levels were observed during preeclampsia, cancer, and neurodegenerative diseases [24,25]. Interestingly, the role of ATP/Mg^2+^ balance in the context of proteostasis maintenance might be supported by studies reporting decreased levels of Mg^2+^ in the brain and cerebrospinal fluid associated with neurodegenerative diseases, including Alzheimer’s and Parkinson diseases, as well as with aging [26,27]. Imbalances in the functional state of the UPS and decreased proteasome activity are frequently observed in these conditions [28,29].

Finally, although ATP does not suppress AMC fluorescence, we cannot exclude the interaction between ATP and the AMC-conjugated peptide substrates that affect the degradation of the latter by the proteasome complex.

In conclusion, we have shown that free ATP affects the degradation of short fluorogenic substrates by the 20S proteasomes. This might impact proteasome activity measurements performed using short fluorogenic substrates, indicating the importance of standardized and carefully selected concentrations of ATP and Mg^2+^ in the reaction mixtures. However, the precise mechanism and possible biological significance of proteasome activity modulation by ATP/Mg^2+^ balance should be addressed in further studies.

## Data Availability

Data is available upon reasonable request.
